# Crypt4GH-JS: securely storing sensitive data online with client-side encryption

**DOI:** 10.1093/bioinformatics/btae763

**Published:** 2025-01-06

**Authors:** Fabienne Thelen, Jannis Hochmuth, Sven Griep, Benedikt Schwab, Alexander Goesmann, Frank Förster

**Affiliations:** Bioinformatics and Systems Biology, Justus Liebig University Giessen, Ludwigsplatz 13-15, Giessen, Hesse, 35390, Germany; Bioinformatics and Systems Biology, Justus Liebig University Giessen, Ludwigsplatz 13-15, Giessen, Hesse, 35390, Germany; Bioinformatics and Systems Biology, Justus Liebig University Giessen, Ludwigsplatz 13-15, Giessen, Hesse, 35390, Germany; Bioinformatics and Systems Biology, Justus Liebig University Giessen, Ludwigsplatz 13-15, Giessen, Hesse, 35390, Germany; Bioinformatics and Systems Biology, Justus Liebig University Giessen, Ludwigsplatz 13-15, Giessen, Hesse, 35390, Germany; Bioinformatics and Systems Biology, Justus Liebig University Giessen, Ludwigsplatz 13-15, Giessen, Hesse, 35390, Germany; Bioinformatics Core Facility, Justus Liebig University Giessen, Ludwigsplatz 13-15, Giessen, Hesse, 35390, Germany

## Abstract

**Motivation and Results:**

Crypt4GH-JS is a browser-ready implementation of the Crypt4GH file encryption standard written in JavaScript. While having minimal to no impact on data upload and download throughput this library enables on-the-fly encryption of arbitrary data in web applications, regardless of whether on the client or server side. As development moves more and more toward cloud-native applications, this library represents a significant step forward for flexible data security in the context of opaque cloud storage systems.

**Availability and implementation:**

Crypt4GH-JS can be installed via Node Package Manager (https://www.npmjs.com/package/crypt4gh_js) or through its public GitHub Repository (https://github.com/fathelen/crypt4ghJS), where the source code is available. Crypt4GH-JS can be tested in the browser using our demonstration website, which can be found at: https://fathelen.github.io/crypt4ghJS/.

## 1 Introduction

The storage of sensitive data is a significant concern in contemporary times, particularly within web-based data management platforms. Thus these have to prioritize data security. One of the most effective measures is to encrypt and decrypt data on the client-side before it leaves the secure user environment.

The Global Alliance for Genomics and Health (GA4GH) (Rehm *et al.* 2021) has developed a standard file container format focusing on biological data named Crypt4GH (Senf *et al.* 2021). It ensures secured storing and sharing of sensitive data, using envelope encryption. Envelope encryption is an encryption protocol using a two layer encryption system: symmetric keys for the inner layer and asymmetric keys for the outer layer. Current implementations for de- and encryption according to the Crypt4GH format are focusing on nonweb-based applications, e.g. applications written in Python (EGA-Archive 2024a, https://github.com/EGA-archive/crypt4gh) or Rust (EGA-Archive 2024b, https://github.com/ega-archive/crypt4gh-rust). In addition to standalone software, web service-based developments have gained increasing significance. Notably, the Oxford Academic journal Nucleic Acids Research began publishing its annual Web Server Issue 21 years ago to reflect this growing importance (Editorial 2003, Sale and Stoddard 2024, Seelow 2024). Such services mostly benefit from libraries that are written in a web programming language, but to this day no client-side Crypt4GH library is available.

Our aim was to tackle the problem of missing software for secure storage of sensitive data by implementing client-side encryption following the Crypt4GH standard using a web-based programming language. Hereby, we wanted to minimize the impact on data transfer duration to avoid compromising the user experience.

Herein, we present our web compatible solution, Crypt4GH-JS, which has been implemented in JavaScript and is therefore applicable to all modern browsers. Crypt4GH-JS implements all features developed for Crypt4GH (Senf *et al.* 2021) with a negligible impact on up- and download time. Through the integration of Crypt4GH-JS into web-based data management tools, sensitive data is encrypted and decrypted on the clients site. Therefore, it is safe to upload, store, and share sensitive data in web-based data management tools using Crypt4GH-JS.

To showcase the web usability of Crypt4GH-JS, we developed a demonstration website (Thelen 2024a, https://fathelen.github.io/crypt4ghJS/). On this website the key generation as well as encryption and decryption can be tested.

## 2 Materials and methods

Crypt4GH-JS uses the specifications given by the GA4GH File Encryption Standard (samtools 2024, https://github.com/samtools/hts-specs/blob/master/crypt4gh.pdf), as well as parts of the Python crypt4gh implementation codebase (EGA-Archive 2024a). To implement Crypt4GH-JS we had to work out a concept, that allows using this specification in the browser. Therefore two main changes were made compared to the Python implementation: First the transformation from a buffer-based reading and writing approach to a stream-based approach and second the transformation of the edit list for decryption from one edit list for the complete data to one edit list for each streamed 65 536 B chunk of data.

### 2.1 Stream-based approach

To minimize the web storage and enable the user to upload huge data files to web-based data management systems, we decided to use a stream-based approach. Uploaded files are streamed until chunks of 65 536 Bytes are filled as specified in the GA4GH File Encryption Standard. Note that final chunks might be smaller than 65 536 Bytes and are not padded to the specified block size. These chunks are encrypted afterwards in concurrence with the block based approach of the Python implementation. The header and the individual chunks are encrypted separately on the client-side and can then be send, already encrypted, to the server. For the decryption we used the same concept, ciphertext chunks of 65 564 Byte are streamed and decrypted separately on the client-side. The difference in size (28 Byte) between cipher- and plaintext is caused by the nonce and Message Authentication Code (MAC) required for the specified encryption method chacha20-ietf-poly1305, according to the GA4GH File Encryption Standard (samtools 2024).

### 2.2 Transformation of edit list

The edit list is a concept of Crypt4GH (Senf *et al.* 2021), which allows to specify parts of files that can be decrypted by a certain user. It contains alternately one after the other the number of bytes that should be skipped and the number of bytes that should be decrypted. Due to the stream-based approach, we had to recalculate the corresponding bytes to the position in the currently streamed chunk. A detailed explanation for the stream-based approach and the transformation of the edit list can be found at: (Thelen 2024d, https://github.com/fathelen/crypt4ghJS/blob/master/Stream_based_approach.md).

### 2.3 Details for demonstrator

The Crypt4GH-JS demonstration website (Thelen 2024a) was built using GitHub Pages. GitHub Pages allows hosting a website directly from the corresponding GitHub repository. In this case it is based on the Crypt4GH-JS Repository (Thelen 2024b, https://www.npmjs.com/package/crypt4gh_js). The demonstration website includes a three-step tutorial; to learn how to create a keypair, encrypt data and decrypt data.

### 2.4 Benchmarking

Crypt4GH-JS is designed for the client-side processing of Crypt4GH container files. Therefore, the library should run in the user’s browser. In this scenario, the bandwidth with which the data is read or written is limited by the network: either unencrypted data is read on the user’s side in order to be encrypted in the browser and then securely stored on a server, or encrypted data is retrieved by the browser via the network and is decrypted there. In order to determine the maximum data throughput that can be achieved during encryption and decryption, we have therefore limited the speed of the incoming data stream to values that correspond to common network speeds: 20, 50, 100, 250, 500, 1000 Mbit/s. In addition, we wanted to investigate the impact of the file size and therefore examined seven different file sizes. For each parameter setting, we encrypted and decrypted the dataset and compared this throughput against the runtime of the plaintext transfer. Each measurement was taken ten times after two warm-up runs. Therefore, we utilized the software hyperfine (Peter 2023, Version 1.17.0, https://github.com/sharkdp/hyperfine). The runtime was recorded and the maximum throughput was limited by the software pipeViewer (Wood (a-J Wood) 2024, Version 1.7.24, https://codeberg.org/a-j-wood/pv). For our analyses the file size for the parameter set was divided by the median of the runtimes to calculate the achieved throughput in Mbit/s.

## 3 Results

### 3.1 Implementation

Crypt4GH-JS is written in JavaScript. It is available as Node Package Manager (NPM) package (Thelen 2024b) and from the GitHub Repository Crypt4GH-JS (Thelen 2024c, https://github.com/fathelen/crypt4ghJS). It allows the handling of data stored in the Crypt4GH (Senf *et al.* 2021) standard file container format inside browsers. Encryption primitives were imported from a number of external NPM modules (for details see the dependency list). Crypt4GH-JS supports the encryption of plaintext, as well as the decryption of cyphertext. Moreover, it enables users to generate asymmetric encryption keys. Finally, all proposed features of the Crypt4GH container file system are supported including edit lists.

### 3.2 Performance

**Figure 1.**
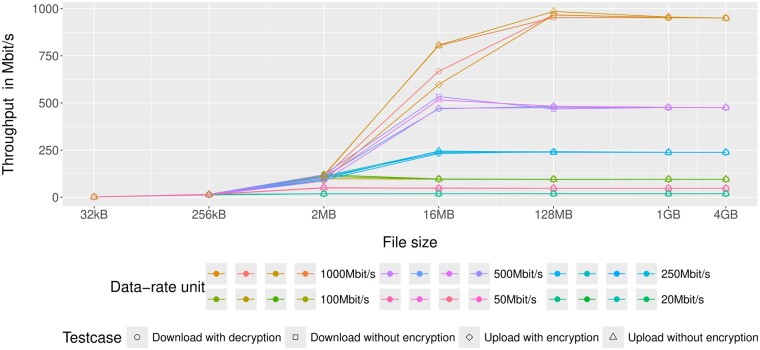
Overview of the benchmarking results. The *x*-axis shows the tested file sizes. The *y*-axis shows the throughput in Megabit per second. The six tested data-rate units: 1000, 500, 250, 100, 50, and 20 Mbit/s are tested for each of the four testcases: download with decryption (circles), download without decryption (squares), encryption with encryption (rhombus), and upload without encryption (triangles). In total, we received 168 cases for the benchmarking: The benchmarking was done with hyperfine (Peter 2023, Version 1.17.0, https://github.com/sharkdp/hyperfine) and pipeViewer (Wood (a-J Wood) 2024, Version 1.7.24, https://codeberg.org/a-j-wood/pv) to track the process and limit the data-rate unit, for two warm-up runs and ten benchmarking runs. The results are calculated from the files size in Megabit divided by the median of the ten benchmarking runs to receive the throughput in Megabit per second per case (*y*-axis).

To test the effective throughput of Crypt4GH-JS we compared uploads with and without encryption as well as downloads with and without decryption. The normalized throughput in Mbit/s for all parameter settings is plotted (Fig. 1). The achieved throughput is almost independent from the type of operation, throughout all file sizes and maximum throughput limits. Therefore, the overhead required by our de- and encryption operations are negligible.

In addition, the results indicate a saturation of the resulting throughput. This saturation occurred instantaneously for smaller data rate limits (100, 50, and 20 Mbit/s, Fig. 1). 250 and 500 Mbit/s saturated at a file size of 16 MB and finally the saturation for 1000 Mbit/s started at 128 MB file size. This saturation is caused by the data rate limit applied, thus the impact of data de- or encryption is marginal in comparison to the bandwidth limitation. Crypt4GH-JS costs minimal to no additional upload and download time for the user.

## 4 Discussion

We could show, that using Crypt4GH-JS in the browser to upload and encrypt or download and decrypt data, is slightly more time-consuming than uploading and downloading without encryption/decryption. Our implementation allows the saturation of a typical network interface, thus a secured data transfer does not result in a loss of transfer speed and will be hardly recognized by the user.

In contrast to other Crypt4GH implementations such as Python or Rust, Crypt4GH-JS demonstrated a lower performance by about 83% for encryption and 50% for decryption in comparison to the Python implementation (further details see Supplementary Material). This is due to the limitations of JavaScript, especially in the browser. But as we have shown, this increased runtime compared to other implementations, is not limiting in the envisioned use-case and therefore negligible. Crypt4GH-JS is the only implementation available to use the Crypt4GH standard file container format in the browser, where the limiting factor is the maximal given data-rate unit.

Finally, Crypt4GH-JS offers the ability to handle edit lists as specified by the Crypt4GH standard, which is currently not supported by the implementations in Python or Rust. This is ensured by applying the example postulated in the Crypt4GH standard specification for an CRAM sequencing file (samtools 2024, further details see Supplementary Material). Nevertheless we ensured that the tools are compatible.

## 5 Conclusion

We developed a browser-ready Crypt4GH implementation that is fully compatible to the Python implementation and fulfills all prescribed parameters for the Crypt4GH container file format. Furthermore, Crypt4GH-JS costs minimal to no additional upload and download time for the user, and can be easily integrated into both, new and existing projects.

## Supplementary Material

btae763_Supplementary_Data

## Data Availability

The source code of Crypt4GH-JS is available at our GitHub Repository https://github.com/fathelen/crypt4ghJS. In addition, the installation is possible as an NPM package from https://www.npmjs.com/package/crypt4gh_js. The demonstration site is hosted by GitHub Pages at https://fathelen.github.io/crypt4ghJS/.
