# Supplementary material for: Crypt4GH-JS: securely storing sensitive data online with client-side encryption
Source: Bioinformatics. 2025 Jan 6;41(1):btae763. doi: 10.1093/bioinformatics/btae763 (PMC11771768; doi:10.1093/bioinformatics/btae763)
Supplement: btae763_Supplementary_Data [file btae763_supplementary_data.zip › 3eef7_supplement.pdf]

This document serves as the supplementary material for the paper "`Crypt4GH-JS`: Securely storing sensitive data online with client-side encryption". It provides a more detailed presentation and verification of the benchmark results and includes an in-depth explanation of how our edit lists fulfill the given specifications.

Benchmark Python vs. `Crypt4GH-JS`

Python runtime evaluation

To compare the runtimes of the Python implementation and `Crypt4GH-JS` we encrypted and decrypted the same files seperatly with both tools.

In the first step we had to create keypairs for this comparison:

secret-key files

Bob:

```
-----BEGIN CRYPT4GH PRIVATE KEY-----
YzRnaC12MQAEbm9uZQAEbm9uZQAgh1ozK/S+7fSJj5omNY8DBqv5refuR66O9b8jSq6/iS4=
-----END CRYPT4GH PRIVATE KEY-----
```

Alice:

```
-----BEGIN CRYPT4GH PRIVATE KEY-----
YzRnaC12MQAEbm9uZQAEbm9uZQAgGwnxf1JmGQx2tTYVr9wjyvx/7/6lGDFF6JQlBeOOb40=
-----END CRYPT4GH PRIVATE KEY-----
```

public-key files

Bob:

```
-----BEGIN CRYPT4GH PUBLIC KEY-----
kXiGXMD/RhC40ZvJ+Xf8tUmhOgJZ5Qe7Q9xHtzQi8Qo=
-----END CRYPT4GH PUBLIC KEY-----
```

Alice:

```
-----BEGIN CRYPT4GH PUBLIC KEY-----
J1jUwKdtm5YpFA1PAVGe/J1pFg9Akln+BJl9rEn1e34=
-----END CRYPT4GH PUBLIC KEY-----
```

Second, we created three files in the size range: 32 kB, 2 MB and 1 GB with the following command:

```
echo 6494a2986b32e324f20a68a2c75ad3d1244de346c6d1b246632c64223bae6d5d| \
   openssl enc -aes-128-ctr -in /dev/zero -pass stdin -nosalt | \
   dd if=/dev/stdin of=32kb bs=4k count=8

echo 6494a2986b32e324f20a68a2c75ad3d1244de346c6d1b246632c64223bae6d5d| \
   openssl enc -aes-128-ctr -in /dev/zero -pass stdin -nosalt | \
   dd if=/dev/stdin of=2mb bs=4k count=512

echo 6494a2986b32e324f20a68a2c75ad3d1244de346c6d1b246632c64223bae6d5d | \
   openssl enc -aes-128-ctr -in /dev/zero -pass stdin -nosalt | \
   dd if=/dev/stdin of=1gb bs=4k count=256k
```

In the third Step, we did the becnhmarking for the Python implementation and cryptghjs with the following commands (in this example with the 1 GB input file):

```
hyperfine --warmup 2 --runs 10 --export-json SUMMARYFILE1gb \
   'crypt4gh encrypt --sk alice.sec --recipient_pk bob.pub < 1gb > 1gb.c4gh |
   wc -c'

hyperfine --warmup 2 --runs 10 --export-json SUMMARYFILE1gb \
   'node bench.js |
   wc -c'
```

For the runtime comparison we tested the 3 file sizes for encryption and decryption with both tools. Before each benchmark we used two warm-up runs followed by ten recorded runs.

Table S1: Runtime comparison between Python and `Crypt4GH-JS` implementation for encrypting and decrypting of different file sizes. The factor indicates how much slower the `Crypt4GH-JS` implementation runs compared to the Python implementation.

File size	Encryption			Decryption		
	Python [s]	Crypt4GH-JS [s]	Factor	Python [s]	Crypt4GH-JS [s]	Factor
32 kB	0.0471	0.0863	1.83	0.0466	0.0753	1.61
2 MB	0.0517	0.0959	1.85	0.0494	0.0765	1.54
1 GB	1.7875	3.2319	1.8	1.7643	2.3833	1.35

Proving the conformity of input and output file content

To ensure that the decrypted files are identical to the original plaintext files, we calculated SHA256 hash sums for the plaintext file, the decrypted Python file and the decrypted `Crypt4GH-JS`.

For this test we used the following command:

```
shasum -a 256 PLAINTEXT PYTHONDECRYPTION CRYPT4GH-JSDECRYPTION
```

Our results show, that both tools encrypt and decrypt the data without changing the original data:

32 kB:

```
454c8c2d4b48fdc6f119f735a79cc9d851034e76f757ac200a1ed1315b4d7ba2 32kb (Plaintext)
454c8c2d4b48fdc6f119f735a79cc9d851034e76f757ac200a1ed1315b4d7ba2 32kbdec (Crypt4GH-JS)
454c8c2d4b48fdc6f119f735a79cc9d851034e76f757ac200a1ed1315b4d7ba2 32kbdec (Python)
```

2 MB:

```
d011dad33a5129dffd61ff357678296adca28eec7c8de5b68f0f1ecbd4e33a07 (Plaintext)
d011dad33a5129dffd61ff357678296adca28eec7c8de5b68f0f1ecbd4e33a07 (Crypt4GH-JS)
d011dad33a5129dffd61ff357678296adca28eec7c8de5b68f0f1ecbd4e33a07 (Python)
```

1 GB:

```
c6136524012555e3835957758eefd6961d3b6953b0b2f5658c02b38e4c89cd3c (Plaintext)
c6136524012555e3835957758eefd6961d3b6953b0b2f5658c02b38e4c89cd3c (Crypt4GH-JS)
c6136524012555e3835957758eefd6961d3b6953b0b2f5658c02b38e4c89cd3c (Python)
```

Edit list

To ensure compatibility between the Crypt4GH specifications and `Crypt4GH-JS` we tested the edit list example from the Crypt4GH specification document.
For this we:

1. Download `ftp://ftp.sra.ebi.ac.uk/vol1/run/ERR243/ERR2436651/21541_1%234.cram`
2. Create a new keypair on `https://fathelen.github.io/crypt4ghJS/`
3. Encrypt the downloaded file on our demo website with the edit list '0, 7853, 71721, 307929, 51299, 38' (given in the Crypt4GH specification)
4. Decrypt the resulting c4gh file
5. Compare the number of bytes in the decrypted file, with the number of "keeping bytes" in the edit list.

```
Sum of keeping bytes: 7853+307929+38 = 315820
Bytes in decrypted file tested with 'cat cramdec | wc -c'  are 315820
```

To also visualize how the edit list for web use looks like:

```
1 => [ 0n, 7853n ],
2 => [ 14038n, 51498n ],
3 => [ 0n, 65536n ],
4 => [ 0n, 65536n ],
5 => [ 0n, 65536n ],
6 => [ 0n, 59823n ],
7 => [ 45586n, 38n ]
```
